# PRRSV Vaccine Strain-Induced Secretion of Extracellular ISG15 Stimulates Porcine Alveolar Macrophage Antiviral Response against PRRSV

**DOI:** 10.3390/v12091009

**Published:** 2020-09-10

**Authors:** Hongbin Liu, Bingjun Shi, Zhigang Zhang, Bao Zhao, Guangming Zhao, Yijing Li, Yuchen Nan

**Affiliations:** 1 College of Animal Medical Sciences, Northeast Agricultural University, Harbin 150030, China; hswzp01@163.com; 2 Department of Preventive Veterinary Medicine, College of Veterinary Medicine, Northwest A&F University, Yangling 712100, China; 13333482298@163.com (B.S.); zhangzhigang123@163.com (Z.Z.); 3 Shaanxi Animal Disease Control Center, Xi’an 710054, China; joben71558@163.com (B.Z.); dearzgm@126.com (G.Z.)

**Keywords:** interferons, PRRSV, ISG15, macrophage, interferons-stimulated genes, cytokine

## Abstract

Porcine reproductive and respiratory syndrome virus (PRRSV) has disrupted the global swine industry since the 1980s. PRRSV-host interactions are largely still unknown but may involve host ISG15 protein. In this study, we developed a monoclonal antibody (Mab-3D5E6) specific for swine ISG15 (sISG15) by immunizing mice with recombinant sISG15. A sandwich enzyme-linked immunosorbent assay (ELISA) incorporating this sISG15-specific Mab was developed to detect sISG15 and provided a lower limit of sISG15 detection of 200 pg/mL. ELISA results demonstrated that infection of porcine alveolar macrophages (PAMs) with low-virulence or attenuated PRRSV vaccine strains induced intracellular ISG15 expression that was independent of type I IFN production, while PAMs infection with a PRRSV vaccine strain promoted extracellular ISG15 secretion from infected PAMs. Conversely, the addition of recombinant sISG15 to PAMs mimicked natural extracellular ISG15 effects whereby sISG15 functioned as a cytokine by activating PAMs. Once activated, PAMs could inhibit PRRSV replication and resist infection with PRRSV vaccine strain TJM. In summary, a sandwich ELISA incorporating homemade anti-ISG15 Mab detected ISG15 secretion induced by PAMs infection with a PRRSV vaccine strain. Recombinant ISG15 added to cells exhibited cytokine-like activity that stimulated PAMs to assume an anti-viral state that enabled them to inhibit PRRSV replication and resist viral infection.

## 1. Introduction

Porcine reproductive and respiratory syndrome virus (PRRSV) is a positive-sense, single-stranded, enveloped RNA virus which belongs to the genus *Porartevirus* [1,2]. According to the latest classification system, there are two species within the *Porartevirus*: *PRRSV-1* and *PRRSV-2* [1,2]. Isolates from *PRRSV-1* and *PRRSV-2* share only 60% nucleotide sequence identity and exhibit serotype differences [3,4]. PRRSV infection in vivo is highly restricted to immune cells of monocyte/macrophage lineage origin, such as pulmonary alveolar macrophages (PAMs) [5,6], macrophages in lymph organs, peritoneal macrophages in blood, and progenitor cells in bone marrow [7,8,9,10]. Generally, PAMs in the lungs are considered the major target of PRRSV infection in vivo [5,6]. Typical immune features in hosts after PRRSV infection include persistent viremia, strong inhibition of innate immunity (IFN-α/β, TNF-α, IL-1β), dysregulation of NK cell function, rapid induction of non-neutralizing antibodies, delayed onset of viral neutralizing antibodies, and CD8+ T-cell mediated cytotoxicity, as well as rapid induction of regulatory T-cells [6,11,12].

Interferons (IFNs) are a large group of secreted cytokines that play indispensable roles in antiviral innate immunity. The type I IFNs, which include IFN-α, IFN-β, IFN-ε, IFN-κ, and IFN-ω [13,14], constitute the largest IFN family [15]. All type I IFNs (hereafter referred to as IFNs) bind to ubiquitously expressed cell membrane-bound heterodimers composed of IFN-α receptor 1 (IFNAR1) and 2 (IFNAR2), then activate JAK/STAT pathways to trigger expression of IFNs-stimulated genes (ISGs) [16]. ISGs have diverse functions, such as amino acid and lipid metabolic regulation, antigen processing and presentation, cellular signaling, transcriptional regulation, ubiquitination, apoptosis, host defense, immune modulation, inflammation, and tumor suppression [17]. The most important ISGs are IFNs effector molecules possessing antiviral activities, such as ISG15, MX1, members of the 2′-5′ oligoadenylate synthetase (OAS) family, and RNase L.

ISG15, one of the first ISGs to be discovered, has been recognized as a homologue for ubiquitin (Ub) [18]. Mature ISG15 is generated by removing of eight amino acids located at the C-terminus of a precursor protein. The mature ISG15 protein shares an identical sequence motif, LRLGG, with the C-terminus of mature Ub [19]. Unlike other Ub homologues (e.g., SUMO), ISG15 has not yet been found in yeast, insects, plants, or other lower organisms, implying that it has a highly specialized function in vertebrates [20]. In addition to its putative intracellular function, researchers have long known that ISG15 could be secreted from monocytes and lymphocytes exhibited cytokine-like activities [21,22]; such activities included stimulation of NK cell activation, induction IFN-γ production of T-cell in a dose-dependent manner [21], and neutrophil chemotactic factor-like activity [22,23]. A recent study demonstrated that extracellular ISG15 activated PBMC via triggering of the LFA-1 integrin receptor (CD11a), leading to enhanced secretion of IFN-γ and IL-10 [24]. However, the role of secreted ISG15 in swine remains unclear.

Researchers have made tremendous efforts to elucidate the interaction between PRRSV and the host innate immune response in order to better understand viral pathogenesis. Previous studies have established that the PRRSV genome encodes several IFN antagonists that block either IFN induction or IFN-activated JAK/STAT signaling [25,26,27], such as NSP1α, NSP1β [28], NSP2 [29,30], NSP4 [31], and NSP11 [32]. Notably, PRRSV NSP2 contains a papain-like protease domain (PLP2), which belongs to the ovarian tumor domain-containing superfamily of deubiquitinating enzymes and therefore is capable of counteracting ISG15 conjugation with cellular proteins [33]. However, the cytokine-like role played by secreted ISG15 during the PRRSV infection has remained elusive, due to the unavailability of anti-sISG15 antibody.

In this study, we first created a novel monoclonal antibody (Mab) that recognized swine ISG15 (sISG15). Next, we incorporated the Mab into a sandwich ELISA (also developed here) to detect secreted sISG15 in PRRSV-infected porcine alveolar macrophage (PAM) culture supernatants. The results demonstrated that the virulent PRRSV infection of PAMs led to a strong inhibition of both sISG15 expression and secretion, while the low-virulence PRRSV strain or attenuated vaccine stain induced sISG15 expression independent of IFN induction in PAMs. Notably, infection of PAMs with a PRRSV vaccine strain TJM-F92 (TJM) resulted in the secretion of extracellular sISG15. Furthermore, the addition of recombinant sISG15 to PAMs prior to inoculation with a virulent PRRSV strain appeared to induce an antiviral state in the PAMs and strongly inhibit viral replication in PAMs. Taken together, our data suggest that ISG15 may function as an antiviral cytokine in PAMs such that the induction of secreted (extracellular) sISG15 is related to the virulence phenotype of PRRSV strain in vivo.

## 2. Material and Methods

### 2.1. Cells, Viruses, Chemicals, and Plasmids

MARC-145 cells and 3D4/21 ATCC^®^ CRL-2843 cells were maintained in Dulbecco’s Modified Eagle Medium (DMEM; Biological Industries, Israel) supplemented with 10% FBS (Biological Industries). Porcine alveolar macrophages (PAMs) were collected from four-week-old PRRSV-negative pigs as previously described [34]. PAMs were maintained in a RPMI 1640 medium (Biological Industries) supplemented with 10% FBS (Biological Industries).

PRRSV virus isolates used in this study included VR2332 (GenBank: EF536003.1) and highly pathogenic PRRSV (HP-PRRSV) isolates, including SD16 (GenBank: JX087437.1), HUN4 (GenBank: EF635006.1), JXA1 (GenBank: EF112445.1), NADC30-like HNhx (GenBank: KX766379), and HP-PRRSV-derived modified live virus (MVL) vaccine strain TJM-F92. All PRRSV isolates were used to inoculate PAMs or MARC-145 cells at multiplicity of infection (MOI) values as indicated. Median tissue culture infectious doses (TCID_50_) of all PRRSV isolates (except NADC30-like isolate HNhx) were titrated in MARC-145 cells as previously described [35]. Inoculation and titration of the NADC30-like isolate HNhx was conducted in PAMs. Recombinant porcine IFN-α used in this study was purchased from the R&D system (Minneapolis, MN, USA).

### 2.2. RNA Isolation, Reverse Transcription, Plasmids Construction, and qPCR

Total RNA was isolated from PAMs using RNAiso Plus (TaKaRa, Dalian, China) and reverse transcribed using the PrimeScript^®^ RT reagent Kit (TaKaRa) according to the manufacturer’s instructions. The cDNA sequence of swine ISG15 (sISG15) was cloned from cDNA of PAMs using Q5^®^ High-Fidelity DNA Polymerase (New England Biolabs, Ipswich, MA, USA) and ligated to pET-28a prokaryotic expression vector with a 6×His-tag fused to the N-terminus. The constructed pET-28a-6×His-sISG15 vector was confirmed via DNA sequencing then was transformed into *Escherichia coli* strain BL21 (DE3) for protein expression analyses. Primers used for cloning and their corresponding sequences are listed in Table 1.

For the qPCR analysis of relative gene expression, reverse transcription and qPCR were conducted using a PrimeScript RT reagent kit (TaKaRa), as previously described with indicated primers [36]. Transcripts of GAPDH were also amplified and used to normalize the total RNA input. The primers and corresponding sequences used for qPCR amplification are also listed in Table 1. Relative quantification of target genes was calculated using the 2^−ΔΔCt^ method.

### 2.3. Expression of Recombinant Swine ISG15

The pET-28a vectors encoding sISG15 were transformed into *E. coli* strain BL21 (DE3) and cultured in a LB medium at 37 °C until they were induced with 0.5 mM isopropyl β-D-thiogalactoside (IPTG) at 25 °C. After IPTG induction, bacterial cells were collected and resuspended in a cell lysis buffer (50 mM Tris–HCl (pH 7.5), 150 mM NaCl, 1 mM EDTA, 1 mM AEBSF (4-benzenesulfonyl fluoride hydrochloride), and 5% glycerol) for sonication. Cell debris was removed and the supernatant containing recombinant 6× His-tagged sISG15 was further purified using Ni+ affinity chromatography (Transgene, Beijing, China). After elution of His-tagged protein using an elution buffer (50 mM Tris (pH 7.5), 150 mM NaCl, and 250 mM imidazole) the purified protein preparation was dialyzed against PBS. Purified recombinant proteins were quantified using the BCA Protein Assay Kit (Thermo Fisher Scientific, Waltham, MA, USA) and stored at −80 °C until used for immunizations or to coat ELISA plate wells.

### 2.4. Ethics Statement, Mouse Immunizations, and Cell Fusions

Six-week-old BALB/c mice were obtained from Dashuo Biotech (Chengdu, Sichuan, China). All animal experiments were conducted according to a protocol that had been previously reviewed and approved by the Animal Welfare Committee of Northwest A&F University (Permission code: 2019-YFD-CVM-003, at 7 March 2019). All mice were monitored daily for any clinical signs and every effort was made to minimize the suffering of animals. Euthanasia was performed to achieve a humane end to life according to our protocol.

Mice were immunized with 100 μg of recombinant sISG15 (2 mg/mL in PBS) with an equal volume of adjuvant for a total of three injections separated temporally by two-week intervals. Freund’s complete adjuvant (Sigma-Aldrich, St. Louis, MO, USA) was used for the primary immunization, while Freund’s incomplete adjuvant (Sigma-Aldrich) was used for the remaining injections. Mouse serum was collected weekly for monitoring of serum antibody levels via ELISA. Serum collected before immunization was included as a negative control. After the final immunization, 100 μg of recombinant protein without adjuvant was injected to boost antibody production of mice four days before spleen collection. The mouse with a highest titer of antibodies was used for cell fusion. Cell fusion of spleen cells with S/p20 cells was conducted as previously described using PEG 1500 (Sigma-Aldrich) [37]. Three days after cell fusion, half of the culture supernatant volume was replaced with a fresh medium then the medium was removed and replaced daily for a total of three replacements over three days before ELISA screening. Selected positive hybridoma clones were subjected to subcloning via limited dilution. Isotyping of Mabs was conducted using Mouse Monoclonal Antibody Isotyping Reagents (Sigma-Aldrich) according to the manufacturer’s instructions. Immunization and purification of rabbit anti-sISG15 polyclonal antibodies was conducted by Genscript Biotech Corp. (Nanjing, China).

### 2.5. Enzyme-Linked Immunosorbent Assay (ELISA)

Indirect ELISAs were conducted to evaluate serum antibody levels specific for recombinant sISG15 of immunized mice or in hybridoma supernatants obtained from surviving hybridoma clones after cell fusion. Briefly, purified sISG15 (400 ng) was used to coat 96-well polystyrene microplates (Corning Inc.) in a volume of 100 μL/well PBS (pH 8.0) overnight at 4 °C. Plates were further blocked with 5% skim milk in PBS containing 0.5% Tween 20 (Sigma-Aldrich). The diluted mouse serum or hybridoma supernatant was added to each well and incubated for 1 h at 37 °C followed by washing with PBS containing 0.5% Triton X-100 (Sigma-Aldrich). The binding of antibody to the corresponding antigen was detected using HRP-conjugated goat anti-mouse IgG antibodies (Genscript) and visualized using a 3,3′,5,5′-tetramethylbenzidine (TMB) substrate kit (Tiangen Biotech, Beijing, China). The absorbance of each well was measured using a Victor X5^TM^ Multilabel Plate Reader (Perkin Elmer, Waltham, MA, USA) at a wavelength of 450 nm.

For sandwich ELISA detection of secreted sISG15 in cell culture supernatants from PRRSV-infected PAMs or IFN-treated PAMs, 1 μg of anti-sISG15-Mab-3D5E6 was used to coat ELISA plates to serve as a capture antibody followed by blocking of wells with 5% skim milk in PBS containing 0.5% Triton X-100 (Sigma-Aldrich). Next, samples were added to wells and incubated for 1 h at 37 °C followed by PBS washes containing 0.5% Triton X-100 (Sigma-Aldrich). Next, 100 ng of rabbit anti-sISG15 polyclonal antibody was used to detect Mabs before their use in ELISAs. Interactions of rabbit anti-sISG15 polyclonal antibodies with target proteins were detected using HRP-conjugated goat anti-rabbit IgG antibodies (Jackson ImmuneResearch, West Grove, PA, USA) and visualized using a TMB substrate kit (Tiangen Biotech). A series dilution of recombinant sISG15 was assayed to generate a standard curve for use in sample quantification.

### 2.6. Western Blot (WB) Analysis

Whole cell lysates of PAMs or CRL-2843 cells were harvested in a 1× Laemmli sample buffer (Bio-Rad Laboratories, Hercules, CA, USA) and separated by sodium dodecyl sulfate-polyacrylamide gel electrophoresis (SDS-PAGE), as previously described [38]. Separated proteins were then transferred to PVDF membranes, as previously described [39]. Membranes were probed with homemade anti-sISG15 Mab-3D5E6 or rabbit anti-sISG15 polyclonal antibodies; homemade Mab-6D10 against PRRSV-N protein, or anti-β-tubulin Mab (Transgene). Specific binding between antibodies and targets was detected using HRP-conjugated goat anti-rabbit IgG (Jackson ImmuneResearch) or HRP-conjugated goat anti-mouse IgG (Thermo Fisher Scientific) and visualized using enhanced chemiluminescent (ECL) substrate-based detection (Bio-Rad Laboratories, Hercules, CA, USA). Chemiluminescence signal acquisition was conducted using a ChemiDoc MP Imaging System (Bio-Rad Laboratories) with analysis conducted using the Image Lab software (version 5.1, Bio-Rad Laboratories).

### 2.7. Immunofluorescence Assay (IFA)

CRL-2843 cells with or without added recombinant porcine IFN-α (10 ng) were fixed using 2% paraformaldehyde for 15 min at room temperature, permeabilized with PBS containing 0.5% Triton X-100 (Sigma-Aldrich), then blocked with PBS containing 1% BSA (Sigma-Aldrich). Next, cells were stained with anti-sISG15 Mab-3D5E6. Specific binding between antibody and target was detected using Alexa Fluor^®^ 555-labeled goat anti-mouse IgG (Thermo Fisher Scientific). Cellular nuclei were counterstained with 4′,6-diamidino-2-phenylindole (DAPI; Thermo Fisher Scientific) at 37 °C for 10 min then cells were observed under a Leica DM1000 fluorescence microscope (Leica Microsystems, Wetzlar, Germany). All images were captured and processed using the Leica Application Suite X (version 1.0, Leica Microsystems).

### 2.8. Interferon Bioactivity Assay

Cell culture supernatants of PAMs infected with different PRRSV isolates were harvested and used to treat IFN-deficient VERO cells (also non-permissive for PRRSV) for 24 h without dilution. Next, supernatant-treated VERO cells were further infected with an interferon-sensitive New Castle Disease Virus (NDV) carrying a green fluorescent protein (GFP) reporter gene at a MOI of 0.1, as previously described [40]. After 24 h, NDV-GFP-infected VERO cells were observed under a Leica DM1000 fluorescence microscope (Leica Microsystems) for evaluation of GFP-positive cells. VERO cells without treatment and those infected with NDV-GFP were included as negative controls. Meanwhile, VERO cells treated with 1 unit of porcine IFN-α (defined as the amount resulting in 50% reduction of GFP-positive cells when added to NDV-GFP-infected VERO cells) was included as the IFN-treatment positive control. Recombinant NDV-GFP (LaSota strain carrying virulence F cleavage sites and enhanced GFP) was a gift of Dr. Sa Xiao of Northwest A&F University.

### 2.9. Cell Viability Assay

Cell viability assay were evaluated using the CellTiter-Glo^®^ Luminescent Cell Viability Assay Kit (Promega, Madison, WI, USA) by following the manufacturer’s instructions. Briefly, PAMs were seeded into 24-well plates at a density of 5 × 10^5^ cells/well and cultured for 6 h. Then, recombinant expressed sISG15 at the indicated concentration was used to treat PAMs for another 24 h. Next, PAMs were lysed by using a 1× Passive cell lysis buffer (Promega). Normal PAMs without sISG15 treatment were included as a control. The cell lysate was transferred into a 96-well black polystyrene microplate (Corning Inc, Corning, NY, USA). CellTiter-Glo^®^ reagent was added and mixed with the cell lysate. After incubation for 10 min at room temperature (RT), the luminescence signal was determined with a VICTORX™ ×5 Multilabel Reader (Perkin-Elmer Life and Analytical Sciences, Wellesley, MA, USA).

### 2.10. PRRSV Attachment Assay

PAMs were pre-incubated with the 40 μg sISG15 protein or 40 μg SUMO protein for 24 h at 37 °C or left untreated (control), followed by pre-chilling at 4 °C for 30 min prior to PRRSV inoculation. The PRRSV-JXA1 strain was used to inoculate the PAMs at 0.1 MOI at 4 °C for 1 h to avoid triggering of endocytosis. Subsequently, PAMs were washed three times with pre-chilled PBS to remove unbound virions before being harvested using the RNAiso Plus (TaKaRa) for RT-qPCR to evaluate the viral RNA level from the virions bound.

### 2.11. Statistical Analysis

Results were analyzed using GraphPad Prism version 5.0 (GraphPad Software, San Diego, CA, USA). Statistical significance was determined using either the Student’s *t*-test for comparison of two groups or by one-way analysis of variance (ANOVA) for more than two groups. A two-tailed *p*-value < 0.05 was considered statistically significant.

## 3. Result

### 3.1. Development and Characterization of the Monoclonal Antibody against Swine ISG15

**Figure 1:**
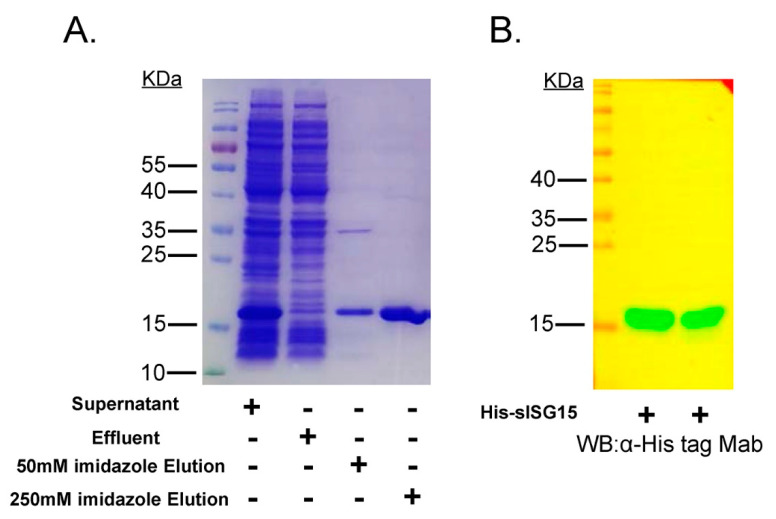
Expression of recombinant swine ISG15 protein (sISG15). (**A**) Supernatant containing soluble 6× His-tagged sISG15 protein after sonication of *E. coli* or analysis of effluent collected after Ni+ column purification of culture supernatants followed by washing and elution of His-tagged protein using an elution buffer of high imidazole concentration. The eluted protein was subjected to SDS-PAGE for analysis of purity of recombinant expressed sISG15. (**B**) Recombinant expressed sISG15 was subjected to SDS-PAGE and Western blotting using the anti-His-tag Mab to confirm successful expression of His-tagged sISG15.

To obtain a monoclonal antibody (Mab) against swine ISG15 (sISG15), the cDNA sequence of sISG15 was ligated to the pET28a vector for expression of recombinant sISG15 with an N-terminal 6× His tag. As demonstrated in Figure 1A, recombinant ISG15 was expressed in a soluble form and was easily purified using Ni+ affinity chromatography. After applying the crude expressed protein preparation to the Ni+ resin column, the column was washed with 50 mM imidazole to remove non-specifically bound proteins. Next, 250 mM imidazole was used to elute highly purified sISG15 His-tagged protein from the column (Figure 1B). The gradient imidazole elution of Ni+ resin column to show the elution peak of sISG15 was demonstrated as Figure S1. After final purification and quantification steps, mice were immunized three times with 100 μg of purified recombinant sISG15 (2 mg/mL in PBS). Then, mouse serum samples were collected weekly to monitor anti-sISG15 antibodies levels. The mouse with the highest anti-sISG15 antibodies titer was used for hybridoma production via cell fusion. Surviving hybridoma cell clones after cell fusion were subjected to ELISA-based screening using a recombinant SUMO-ubiquitin fusion protein (Figure S2) as an unrelated protein control to rule out false-positive results. Supernatants from ELISA-positive hybridomas were further examined for reactivity to recombinant sISG15 via WB or to porcine IFN-α (pIFN-α)-treated cells via immunofluorescence assay (IFA). Only hybridomas with positive results from all three assays (ELISA, WB, IFA) were selected for subcloning.

**Figure 2:**
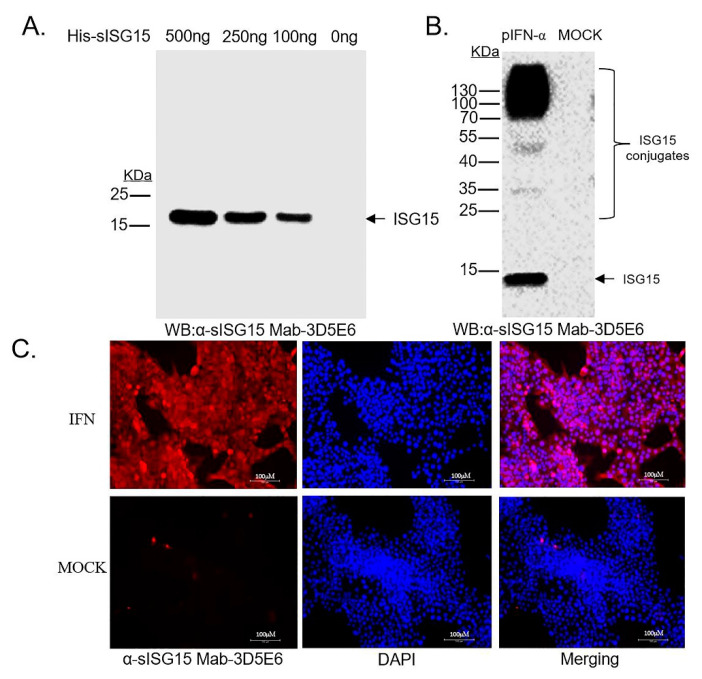
Development of Mab recognizing endogenous swine ISG15. (**A**) Recombinant expressed sISG15 protein with serial dilutions were subjected to Western blotting using the antibody present in hybridoma culture supernatant (clone no. 3D5E6) to detect binding of Mab-3D5E6 to its immunogen. (**B**) Cell lysates from CRL-2843 treated with or without porcine IFN-α (pIFN-α) were harvested and subjected to Western blotting using the hybridoma culture supernatant (clone no. 3D5E6) to detect binding of Mab-3D5E6 to endogenous ISG15 in cell lines of swine origin. (**C**) CRL-2843 cells were either treated with pIFN-α or left untreated for 24 h. After cells were fixed and permeabilized, cells were incubated with Mab-3D5E6 followed by visualization after addition of Alexa Fluor^®^ 555-labeled goat anti mouse IgG (H + L) (Red channel) and counter-staining with DAPI.

After completion of all screening assays and subcloning procedures, hybridoma clone no. 3D5E6 (Mab-3D5E6) was selected. Monoclonal antibody isotyping demonstrated that Mab-2D5E6 was IgG1 (Figure S3). Ultimately, anti-sISG15 Mab-3D5E6 exhibited good reactivity to its immunogen, as demonstrated via the WB analysis (Figure 2A). Meanwhile, both WB and IFA results indicated that Mab-3D5E6 recognized endogenous sISG15 and sISG15-conjugated cellular proteins within CRL-2843 cells when cells were stimulated with pIFN-α (Figure 2B,C), suggesting that anti-sISG15 Mab-3D5E6 was bound with a high affinity to endogenous sISG15 from cells of swine origin.

### 3.2. Establishment of Sandwich ELISA for Detection of Swine ISG15

Since anti-sISG15 Mab-3D5E6 was suitable for IFA, we proposed that it would also be applied as the capture antibody for sandwich ELISA-based detection of extracellular sISG15 from liquid samples (e.g., cell culture supernatants). To confirm this speculation, rabbit anti-sISG15 polyclonal antibodies were prepared for sandwich ELISA detection. To ensure that the rabbit anti-sISG15 polyclonal antibodies only contained antibodies specific for sISG15 and lacked reactivity to other ubiquitin-like molecules, a SOMO-ubiquitin fusion protein was developed (Figure S2) for affinity purification of cross-reactive antibodies from the rabbit anti-sISG15 polyclonal antibodies. Next, after optimization of ELISA procedures, such as Mab coating dose, blocking time, and incubation time, the sISG15 sandwich ELISA was tested using dilutions of recombinant sISG15 to determine assay sensitivity and to generate a standard curve. Based on the standard curve (Figure S4), the detection limit for our homemade sISG15 sandwich ELISA was determined to be 250 pg/mL for liquid samples.

### 3.3. PRRSV Vaccine Strain Induces Secretion of sISG15 in PAMs

**Figure 3:**
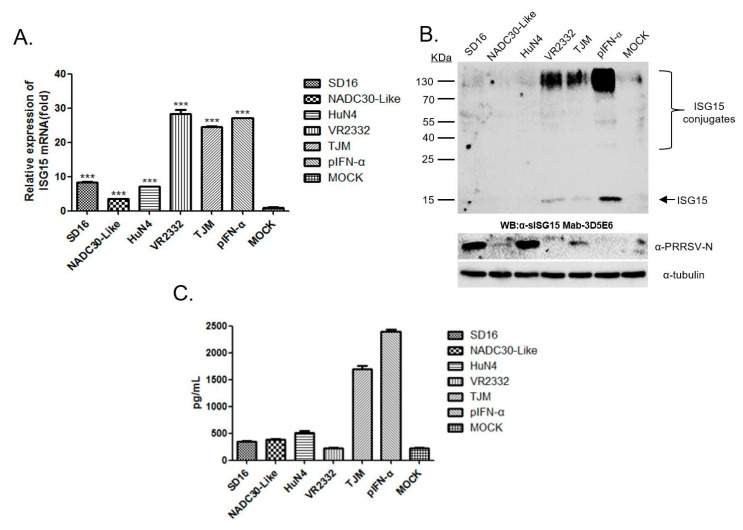
Low-pathogenic porcine reproductive and respiratory syndrome virus (PRRSV) infection-induced expression and secretion of extracellular ISG15 in porcine alveolar macrophages (PAMs). (**A**) PAMs were infected with different PRRSV strains for 24 h then harvested for qPCR to evaluate ISG15 mRNA levels. All experiments were repeated at least three times. Significant differences between indicated groups were marked by *** for *p* < 0.001. (**B**) PAMs were infected with different PRRSV strains at 1 and 2 multiplicity of infection (MOI) values for 24 h then cells were harvested and subjected to Western blot analysis to evaluate ISG15 expression and conjugation activity. PAMs without PRRSV infection or PAMs treated with pIFN-α were included as controls. Replication of PRRSV in PAMs was confirmed using anti-PRRSV-N mAB-6D10 to detect proteins on Western blot membranes. (**C**) Cell culture supernatants from PAMs infected with various PRRSV strains for 24 h were subjected to ELISA to detect levels of extracellular ISG15 secreted by PAMs. Cell culture supernatants from MOCK-infected cells and pIFN-α-stimulated PAMs were included as controls. All data are expressed as mean ± SD representing three independent experiments.

It had been previously reported that the PRRSV infection of PAMs inhibited IFNs-induced activation of the JAK/STAT pathway that subsequently blocked ISGs expression, including expression of ISG15 [25]. However, more recent reports suggested that the PRRSV-based interference with the IFNs-activated JAK/STAT pathway appeared to be PRRSV strain specific, as variable interferences were observed for different strains [41]. Therefore, PAMs were infected with various PRRSV strains for 24 h then harvested for qPCR to evaluate ISG15 mRNA levels. Our results suggested that HP-PRRSV isolates (SD16, HuN4) and the NADC30-like strain (NADC30-like) demonstrated little ability to induce ISG15 expression in PAMs (Figure 3A). Meanwhile, PAMs infected with the *PRRSV-2* prototype strain VR2332 (less virulent than HP-PRRSV and NADC30-like isolates) or HP-PRRSV-derived attenuated MLV vaccine strain TJM-F92 (TJM), induced similar ISG15 mRNA levels in PAMs as pIFN-α-treatment. Moreover, in addition to mRNA levels, intracellular sISG15 protein and ISG15-conjugated cellular proteins in PAMs infected with different PRRSV strains were examined as well. As demonstrated in Figure 3B, sISG15 was detectable in VR2332- and TJM-infected PAMs, but not in PAMs infected with other PRRSV strains. In addition, when replication of PRRSV was measured using anti-PRRSV-N Mab-6D10 probing of the same WB membranes, the results suggested that the PRRSV replication level (as determined by N protein level in infected PAMs) was not directly related to the ISG15 protein level. Thus, ISG15 expression in PRRSV-infected PAMs appears to be strain specific, and only less-virulent PRRSV strains (VR2332 and TJM) were able to induce ISG15 protein expression in infected PAMs. It was also notable that although ISG15 mRNA levels in PAMs infected with VR2332 or TJM were similar to levels in pIFN-α-treated PAMs, ISG15 and ISG15-conjugated protein levels were much lower in PAMs infected with VR2332 and TJM, a result that may be attributed to the putative deubiquitinase activity of the PRRSV-NSP2-OTU domain (Figure 3B).

Next, to examine extracellular levels of ISG15 secreted by PAMs after PRRSV infection, cell culture supernatants from PAMs infected with different PRRSV strains for 24 h were harvested and assayed via our homemade sISG15 sandwich ELISA. The results suggested that the pIFN-α treatment stimulated PAMs to secrete a very high level of extracellular ISG15 that approached 2500 pg/mL. Similar to pIFN-α-treated PAMs, the PRRSV-TJM strain infection of PAMs induced high levels of sISG15 secretion as well, with levels approaching 1700 pg/mL detected in culture supernatants. However, extracellular ISG15 levels secreted by VR2332-infected PAMs surprisingly resembled the low extracellular ISG15 levels observed for MOCK-infected cells, which approached the low limit of detection of the sandwich ELISA. Meanwhile, mRNA and intracellular sISG15 levels in VR2332-infected PAMs resembled levels of TJM strain-infected PAMs or pIFN-α-treated PAMs. Conversely, in PAMs infected with HP-PRRSV or NADC30-like strains, extracellular ISG15 secretion were much lower (300 to 600 pg/mL) than levels determined for TJM-infected PAMs (Figure 3C). Taken together, these results suggest that both low-virulence PRRSV strains (both VR2332 and TJM) could induce expression of intracellular ISG15, while infection of PAMs with PRRSV vaccine strain TJM could additionally induce secretion of extracellular ISG15.

### 3.4. Expression of sISG15 in PAMs Infected by the PRRSV Vaccine Strain Is IFNs-Independent

**Figure 4:**
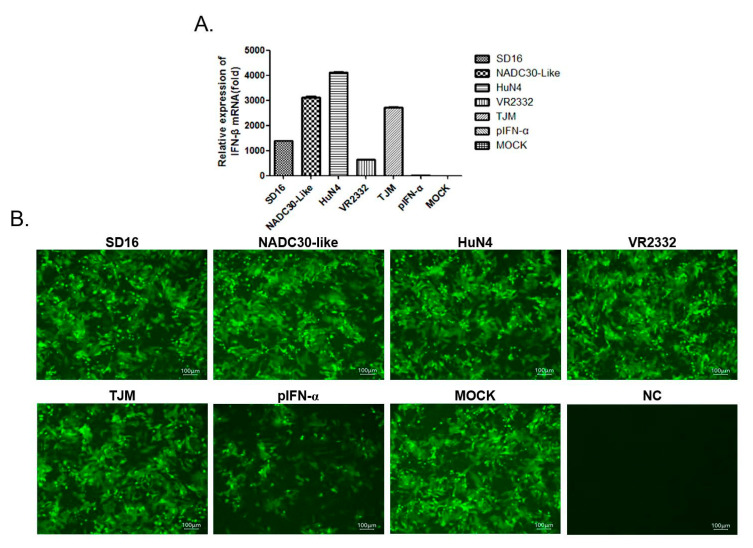
Expression of ISG15 in PRRSV-infected PAMs was IFNs-independent. (**A**) PAMs were infected with heterogeneous PRRSV isolates at 1 MOI for 24 h then cells were harvested for qPCR to evaluate mRNA levels of IFN-β. PAMs without PRRSV infection were included as a control. All experiments were repeated at least three times. (**B**) Cell culture supernatants from PAMs infected with heterogeneous PRRSB isolates were harvested and used to treat VERO cells. After 12 h, VERO cells treated with a cell culture supernatant from PAMs were next infected with IFNs-sensitive NDV carrying GFP as a reporter to monitor IFNs activity in PAMs supernatants. Representative images were captured 24 h after VERO cells were inoculated with NDV-GFP.

It is generally accepted that expression of ISGs, including ISG15, is a consequence of JAK/STAT pathway activation triggered by type I IFNs [42]. However, IFNs-independent ISG15 expression has been observed and reported as well [43]. Meanwhile, PRRSV strains (except for A2MC2 and PRRSV-Con strains) deploy multiple non-structural proteins (NSPs) to antagonize IFNs production, as well as IFNs-activated JAK/STAT signaling in infected cells. Therefore, to determine whether sISG15 induced by a low pathogenic PRRSV infection of PAMs is IFNs-dependent or not, we first examined the transcription level of IFN-β in PRRSV-infected PAMs. The results were not surprising in that all PRRSV strains strongly induced transcriptional activation of IFN-β in the infected PAMs (Figure 4A); which consisted of previous reports showing that PRRSV activated transcription of type I IFNs in PAMs, its natural susceptible cell host [44], but not in MARC-145 cells (a commonly used in vitro PRRSV-permissive cell line). Indeed, PRRSV not only failed to activate transcription of IFN-α/β in MARC-145 cells, but it also suppressed transcription of IFN-β induced by Poly (I:C) [45]. Therefore, it is believed that PRRSV processes post-transcriptional control mechanisms to regulate type I IFNs levels in order to block generation of IFNs proteins in its natural host cell PAMs [44]. Consequently, an NDV-GFP-based IFNs bioactivity assay was conducted using VERO cells to examine IFNs protein levels in cell supernatants of PAMs. Although PAMs and VERO cells did not originate from the same species, VERO cells clearly responded to porcine IFNs but were defective in endogenous IFNs production and non-permissive for PRRSV infection. Nevertheless, these characteristics render VERO cells suitable for use in our IFN bioactivity assay to avoid triggering of pattern recognition receptors (PRRs) in VERO cells by viable PRRSV virions or other PRRs agonists in supernatants generated during PRRSV infection of PAMs.

As demonstrated in Figure 4B, when VERO cells were treated with cell culture supernatants of PAMs infected with different PRRSV strains, no detectable IFN activity was observed, as shown by little reduction of GFP-positive cell. In fact, GFP-positive cell rates were similar across all experimental groups except for cells treated with pIFN-α (Figure 4B). Notably, this result suggested that no IFN proteins were produced by PRRSV-infected PAMs in spite of observed strong activation of IFN-β transcription. Taken together, these data suggest that ISG15 expression observed in PAMs infected with PRRSV-VR2332 and TJM strains was independent of type I IFN production but was directly induced by replication of those virus strains in PAMs.

### 3.5. Extracellular ISG15 Stimulated PAMs Conferred Partial Resistance to PRRSV Infection

**Figure 5:**
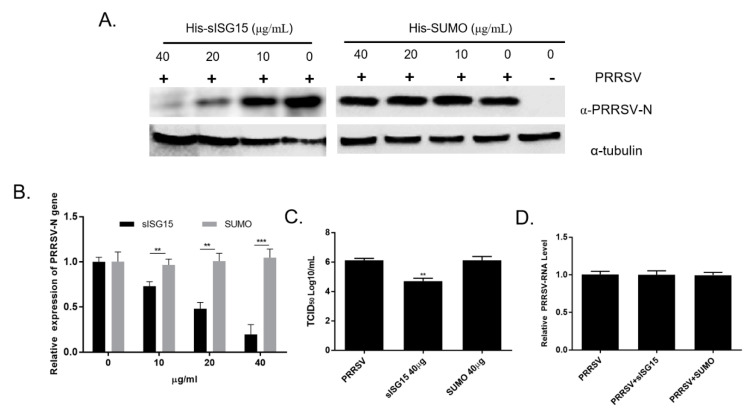
Extracellular ISG15 inhibits PRRSV infection in PAMs. (**A**) PAMs were treated with various dilutions of recombinant sISG15 for 12 h then infected with the PRRSV-SD16 strain at 1 MOI. After 24 h, PAMs were harvested for Western blot analysis then blots were probed with homemade anti-PRRSV-N Mab-6D10 to monitor replication of PRRSV. PAMs treated with recombinant SUMO protein were included as a control. (**B**) PAMs were first treated with either sISG15 or SUMO protein for 12 h then were infected with PRRSV-SD16 strain at 1 MOI. After 24 h of infection, PAMs were harvested for qPCR evaluation of mRNA levels corresponding to PRRSV-N proteins. All experiments were repeated at least three times. Significant differences between indicated groups are marked by ** for *p* < 0.01 or *** for *p* < 0.01. (**C**) Cell culture supernatants from sISG15-treated or SUMO-treated PAMs followed by PRRSV-SD16 infection were titrated to quantify numbers of infectious virus particles in MARC-145 cells. All experiments were repeated at least three times. Significant differences between indicated groups are marked by ** for *p* < 0.01. (**D**) PAMs were pre-incubated with the 40 μg of either sISG15 protein or SUMO protein for 24 h at 37 °C or left untreated (control), followed by pre-chilling at 4 °C for 30 min prior to PRRSV inoculation. The PRRSV-JXA1 strain was used to inoculate the PAMs at 0.1 MOI at 4 °C for 1 h to avoid triggering of endocytosis. After washing cells using pre-chilled PBS to remove unbound virions, the attachment of PRRSV virions was evaluated using qPCR. All experiments were repeated at least three times.

All of the abovementioned data suggest that the attenuated PRRSV vaccine strain TJM could induce upregulation of both intracellular ISG15 production and extracellular secretion of ISG15 by PAMs. Therefore, understanding the biological functions of extracellular ISG15 would be interesting, especially regarding the impact of ISG15 on PRRSV infection. Based on the cell viability assay of PAMs treated with different doses of sISG15, sISG15 was well-tolerated when the concentration of sISG15 was added up to 200 μg/mL (Figure S5). Next, to investigate the role of ISG15 in PRRSV infection, PAMs were treated with various serial dilutions of recombinant sISG15 for 12 h prior to the infection with PRRSV-SD16 strain at 1 MOI. Replication of PRRSV in PAMs was evaluated using a homemade anti-PRRSV-N Mab-6D10 to monitor PRRSV replication. As demonstrated in Figure 5A, PRRSV-N protein levels decreased as the dose of recombinant sISG15 increased, suggesting that the ISG15 treatment of PAMs conferred resistance to infection or inhibition of PRRSV replication. Meanwhile, SUMO protein, another ubiquitin-like protein resembling ISG15, was used as a control protein to treat PAMs as well. Unsurprisingly, PAMs treated with the SUMO protein were still sensitive to PRRSV infection as were non-treated cells (Figure 5A), suggesting that only ISG15 stimulation inhibited PRRSV replication in PAMs.

To further confirm that the inhibitory effect of extracellular ISG15 on PAMs reflected effects on PRRSV replication, PRRSV-N mRNA levels were evaluated via qPCR; the results demonstrated a clear correlation between reduction of PRRSV replication with the increasing sISG15 dose used to treat PAMs (Figure 5B). Moreover, titration of infectious virus particles present in cell culture supernatants from different experimental groups of PAMs further demonstrated that pretreatment of PAMs with recombinant ISG15 strongly inhibited PRRSV replication, leading to a greater than 10-fold reduction in detected infectious virus particles in recombinant sISG15-treated PAMs. Meanwhile, a PRRSV virion attachment assay was conducted to investigate if sISG15 treatment of PAMs inhibit attachment of PRRSV virion. However, based on our result, neither treatment of PAMs using sISG15 or SUMO prevented PRRSV virion attachment to PAMs (Figure 5D). Taken together, these data suggest that extracellular ISG15 may function as a cytokine that stimulates PAMs to mount a response that inhibits PRRSV replication, with extracellular ISG15 secretion possibly attenuating infectivity of PRRSV vaccine strain TJM. In conclusion, this report describes the development of a novel Mab that specifically recognizes ISG15. When used in a sandwich ELISA, this Mab was suitable for use in the evaluation of extracellular ISG15 levels. Notably, ELISA results demonstrated that infection with the PRRSV vaccine strain induced secretion of ISG15 that extracellularly appeared to function as a cytokine that induced PAMs to assume an anti-viral state which resists PRRSV infection.

## 4. Discussion

Interferon-stimulated gen 15 (ISG15), originally identified as an ubiquitin-like molecule, can engage in ISGylation, a process where it undergoes conjugation with protein substrates via the same reversible process employed by ubiquitin [18]. For a long time, ISGylation was thought to be the only functional mechanism for ISG15 production. Unlike ubiquitination, protein ISGylation does not lead to degradation of substrates, as occurs during K48-linked ubiquitination, implying that these processes have distinct functions. However, no universal role has been identified for protein ISGylation, as it is still not known whether ISGylated proteins share a common fate with ubiquitinated proteins or whether fates of individual ISGylated proteins vary [46,47]. Nevertheless, since ISG15 expression is strongly upregulated upon IFNs treatment, ISG15 likely plays an important role in the host innate immune response. Hints regarding ISG15 roles obtained from ISG15-deficient mice demonstrate increased susceptibility of ISG15-deficient mice to influenza virus A and B, Sindbis virus, and HSV-1 [48,49]. Moreover, experiments also show ISG15 targeting of budding of enveloped viruses to inhibit virus replication, such as HIV-1, Ebola, and Chickungunya virus [50]. Notably, interference with host ISGylation processes by viral encoded de-ubiquitin enzymes has been shown to contribute to virus virulence as well. For example, the PRRSV-NSP2 OUT domain was shown to interfere with ISG15 conjugation with cellular proteins [33]. Interestingly, partial relief of the ISG15-antagonistic function of the NSP2-OUT domain via reverse genetics produced a viable recombinant virus with reduced virulence in vivo [33].

It should be noted here that multiple types of immune cells, such as monocytes, neutrophils, and lymphocytes, have been shown to secrete ISG15, with extracellular ISG15 reaching detectable levels in both blood and urine as a reflection of its putative cytokine function [21,22]. ISG15 stimulates proliferation of NK cells and also induces T-cell IFN-γ release in a dose-dependent manner [21]. Although IFN-γ is critical for activating both innate and adaptive immune responses to virus infection in vivo, little is known regarding the true function of extracellular ISG15 or how it participates in activation of intracellular signaling mechanisms. In recent years, the discovery that *ISG15*-deficient patients acquired mycobacterial disease after vaccination with live attenuated Bacillus-Calmette-Guérin (BCG) vaccine provides evidence in support of a role of extracellular ISG15 in IFN-γ induction [51]. Such a role of ISG15 is further supported by the observation that BCG induced IFN-γ secretion from PBMCs was obtained from healthy individuals, but not from PBMCs from ISG15-deficient patients [51]. Within the PBMC population, natural killer (NK) cells and T lymphocytes secrete IFN-γ in response to signals induced by IL-12 and ISG15, with NK cells responding most robustly; which is consistent with their known function as major producers of IFN-γ [24,51]. Meanwhile, leukocyte function-associated antigen-1 (LFA-1, CD11a) has been recently identified as a putative cellular receptor for ISG15 [24]. This role for CD11a is supported by experiments showing that extracellular ISG15 and IL-12 stimulated IFN-γ secretion synergistically in cells with appropriate receptors, while splenocytes from *CD11a*^−/−^ mice did not respond to ISG15. Taken together these results suggest a broader role for ISG15 in cytokine signaling [24]. Nevertheless, since the ISG15 gene is not conserved among mammalian species, the role of extracellular ISG15 in swine species remains unclear.

In this study, a novel monoclonal antibody recognizing endogenous ISG15 in cells of swine origin was generated then used to develop a sandwich ELISA for quantifying ISG15 levels from liquid samples. Notably, treatment of CRL-2843 cells (an immortalized porcine alveolar macrophage cell line) with recombinant IFN-α led to high levels of extracellular ISG15 in cell culture supernatants, suggesting that swine ISG15 was secreted into the extracellular space. Based on our sISG15-specific Mab-based sandwich ELISA, we further examined whether PRRSV infection of PAMs induced upregulation of ISG15 and extracellular secretion of ISG15. Our results suggest that the PRRSV vaccine strain TJM induced secretion of ISG15 to high levels which is similar to IFN-treated PAMs.

As the first characterized member of the ISGs family, numerous studies have shown that ISG15 expression requires cell stimulation with IFNs via either autocrine or paracrine pathways. However, it has been confirmed that the PRRSV infection may lead to inhibition of both IFN-induction and of activation of downstream JAK/STATs signal pathways involving multiple virus-encoded antagonists [26]. Based on the IFNs bioactivity assay used herein, it is notable that all supernatants from PAMs infected with various PRRSV strains (including vaccine strains) demonstrated little IFNs production, which is consistent with our previous observation that the PRRSV replication inhibited IFNs induction (except for infection by two unique strains A2MC2 and PRRSV-Con [52]). Thus, these results suggest that the ISG15 expression in PAMs induced by low-pathogenic PRRSV strains (VR2332 and TJM) should be IFNs-independent. Meanwhile, a recent study demonstrated that pharmacodynamic induction of ISG15 occurred without significant systemic induction of IFNs expression in HBV patients using Vesatolimod, an oral agonist of TLR-7 [43], further supporting that ISG15 expression could be upregulated in an IFNs-independent manner. However, identification of factors involved in the induction of ISG15 expression by low-pathogenic PRRSV strains will require further investigations. Moreover, we must note that although upregulation of ISG15 expression was observed in low-pathogenic PRRSV-infected PAMs, extracellular ISG15 secretion was only detected in PAMs infected with the vaccine strain TJM (a completely attenuated strain). This interesting observation implies that ISG15 secretion may not be an inevitable consequence of ISG15 upregulation after IFNs stimulation or virus infection, and it may require activation of additional cellular pathways. Such pathways may be blocked by PRRSV-VR2332 infection which could be a possible explanation for the inconsistencies of intracellular and extracellular ISG15 levels in VR2332-infected PAMs. Nevertheless, further investigations are needed to reveal pathways involved in the transfer of intracellular ISG15 protein to the extracellular environment.

Finally, the data of this work implicate that ISG15 stimulation resulted in an antiviral state in PAMs. These data not only suggest that macrophages may be potential targets of extracellular ISG15 in swine, but also imply that ISG15 stimulation may play an antiviral role in vivo as well. As for other leukocyte targets of ISG15, it would be interesting to use our homemade ISG15 Mab and sandwich ELISA assay to investigate whether LFA-1 functions as a receptor for ISG15 in swine as it does in mice and humans. In conclusion, our data suggest that an attenuated PRRSV vaccine strain could induce secretion of extracellular ISG15 that functions as a cytokine to induce an anti-viral state that subsequently inhibits PRRSV infection (as observed in vitro in PAMs). The results of this work not only provide novel insights for understanding PRRSV attenuation, but also provide valuable information revealing ISG15 functional roles in swine.

## Figures and Tables

**Table 1:** Primers and corresponding sequences.

Primers	Sequence (5′–3′)	Function
pCold-SUMO-UBI-F	CCCTCGAGCAGATCTTCGTGAAGACCT	Cloning of swine ubiquitin
pCold-SUMO-UBI-R	GCTCTAGATTAGCAGCCACCCCTCAGA	
sISG15-RT-F	AGCAACGCCTATGAGGTCTG	qPCR detection of ISG15
sISG15-RT-R	CCCTCGAAAGTCAGCCAGAA	
GAPDH-F	CCTTCCGTGTCCCTACTGCCAAC	qPCR detection of GAPDH
GAPDH-R	GACGCCTGCTTCACCACCTTCT	
sIFNB-RT-F	AGCACTGGCTGGAATGAAAC	qPCR detection of IFN-β mRNA
sIFNB-RT-R	TCCAGGATTGTCTCCAGGTC	
PRRSV-N-F	ATGCCAAATAACAACGGCAAGCAGC	qPCR detection of PRRSV-RNA
PRRSV-N-R	TCATGCTGAGGGTGATGCTGTG	

## Supplementary Materials

The following are available online at https://www.mdpi.com/1999-4915/12/9/1009/s1. Figure S1: Elution of recombinant sISG15 from the Ni+ resin column using a gradient dose of imidazole to identify the protein elution peak; Figure S2: Expression of SUMO-ubiquitin (SUMO-UB) protein. Ubiquitin cDNA was cloned from PAMs and ligated to pCold-SUMO vector for recombinant expression as a SUMO-UB-fusion protein. SUMO-UB-fusion protein was subjected to SDS-PAGE and WB using anti-His-tag Mab to confirm its expression and purity; Figure S3: Monoclonal antibody isotyping of Mab-3D5E6 specific for swine ISG15; Figure S4: Establishment of standard curve for swine ISG15 sandwich ELISA. One μg of anti-sISG15-Mab-3D5E6 was used to coat plate wells as an ELISA capture antibody followed by blocking of wells with 5% skim milk in PBS containing 0.5% Triton X-100. Next, recombinant sISG15 was added followed by detection using 100 ng of rabbit anti-sISG15 polyclonal antibodies as detection antibodies. Interactions of rabbit anti-sISG15 polyclonal antibodies with target proteins were detected using HRP-conjugated goat anti-mouse IgG antibodies and visualized using TMB substrate; Figure S5: Cell viability assay for PAMs treated with sISG15. PAMs were seeded into 24-well plates at a density of 5 × 10^5^ cells/well and cultured for 6 h. Then, recombinant expressed sISG15 at the indicated concentration was used to treat PAMs for another 24 h. Cell viability of sISG15 treated PAMs were evaluated using the CellTiter-Glo^®^ Luminescent Cell Viability Assay Kit. All experiments were repeated at least three times.
