# Supplementary figures and images for: PRRSV Vaccine Strain-Induced Secretion of Extracellular ISG15 Stimulates Porcine Alveolar Macrophage Antiviral Response against PRRSV
Source: Viruses. 2020 Sep 10;12(9):1009. doi: 10.3390/v12091009 (PMC7551094; doi:10.3390/v12091009)

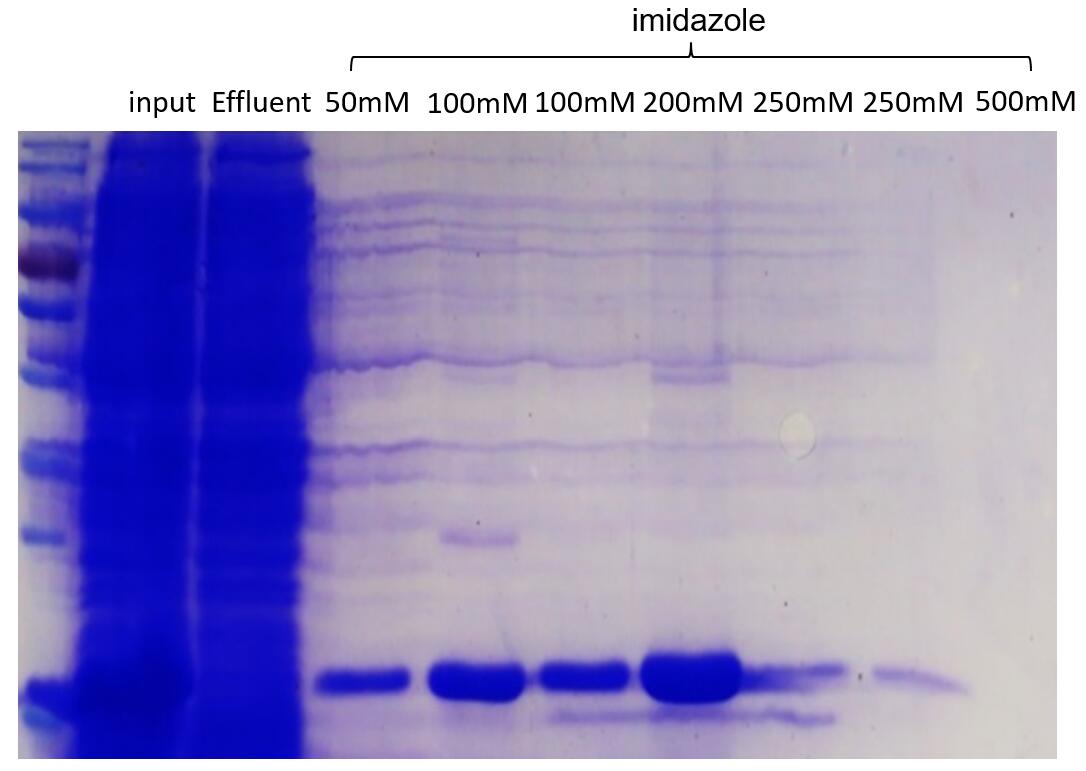

Supplement: Supplementary file 1 [file viruses-12-01009-s001.zip › Supplementary Figures/Figure S1.jpg]

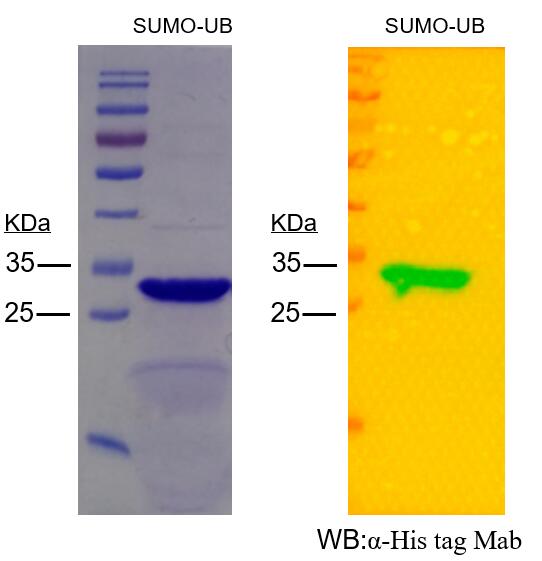

Supplement: Supplementary file 1 [file viruses-12-01009-s001.zip › Supplementary Figures/Figure S2.jpg]

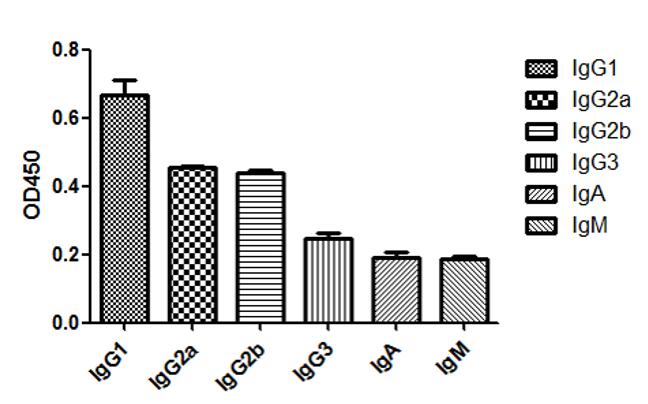

Supplement: Supplementary file 1 [file viruses-12-01009-s001.zip › Supplementary Figures/Figure S3.jpg]

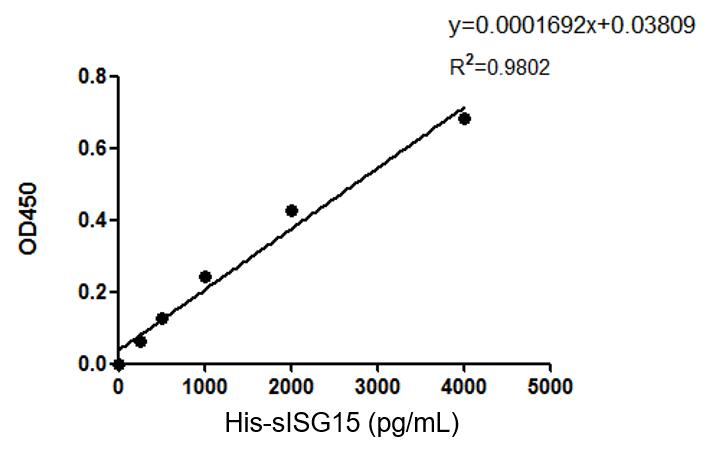

Supplement: Supplementary file 1 [file viruses-12-01009-s001.zip › Supplementary Figures/Figure S4.jpg]

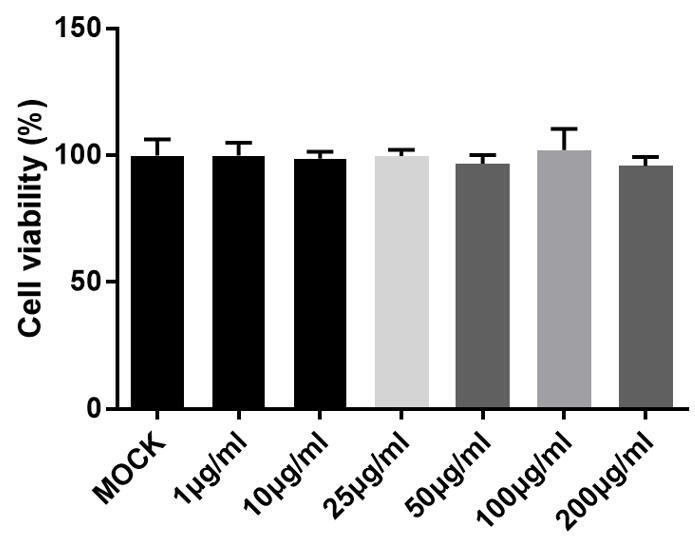

Supplement: Supplementary file 1 [file viruses-12-01009-s001.zip › Supplementary Figures/Figure S5.jpg]
